# Epidemiology and treatment of sepsis at a public pediatric emergency department

**DOI:** 10.31744/einstein_journal/2022AO6131

**Published:** 2022-02-23

**Authors:** Daniela Nasu Monteiro Medeiros, Ana Carolina Cintra Nunes Mafra, Daniela Carla de Souza, Eduardo Juan Troster

**Affiliations:** 1 Hospital Israelita Albert Einstein São Paulo SP Brazil Hospital Israelita Albert Einstein, São Paulo, SP, Brazil.; 2 Hospital Universitário Universidade de São Paulo São Paulo SP Brazil Hospital Universitário, Universidade de São Paulo, São Paulo, SP, Brazil.; 3 Faculdade Israelita de Ciências da Saúde Albert Einstein Hospital Israelita Albert Einstein São Paulo SP Brazil Faculdade Israelita de Ciências da Saúde Albert Einstein, Hospital Israelita Albert Einstein, São Paulo, SP, Brazil.

**Keywords:** Sepsis, Shock, septic, Length of stay, Patient discharge, International Classification of Diseases, Emergency medical services, Epidemiology, Child, Infant

## Abstract

**Objective:**

To describe the clinical characteristics and treatment of children with sepsis, severe sepsis, and septic shock at a pediatric emergency department of a public hospital.

**Methods:**

A retrospective, observational study. The medical records of patients included in the hospital Pediatric Sepsis Protocol and patients with discharge ICD-10 A41.9 (sepsis, unspecified), R57 (shock) and A39 (meningococcal meningitis) were evaluated.

**Results:**

A total of 399 patients were included. The prevalence of sepsis, severe sepsis, and septic shock at the emergency room were 0.41%, 0.14% and 0.014%, respectively. The median age was 21.5 months for sepsis, 12 months for severe sepsis, and 20.5 months for septic shock. Sepsis, severe sepsis, and septic shock were more often associated with respiratory diseases. The Respiratory Syncytial Virus was the most common agent. The median time to antibiotic and fluid administration was 3 hours in patients with sepsis and severe sepsis. In patients with septic shock, the median times to administer antibiotics, fluid and vasoactive drugs were 2 hours, 2.5 hours and 6 hours, respectively. The median length of hospital stay for patients with sepsis, severe sepsis and septic shock were 3 days, 4 days and 1 day, respectively. The overall mortality was 2%.

**Conclusion:**

Sepsis had a low prevalence. Early diagnosis and recognition are a challenge for the emergency care pediatrician, the first place of admission.

## INTRODUCTION

Sepsis is a complex clinical syndrome, resulting from a deregulated response of the body to an infectious insult. It is one of the main causes of death in children, and the final pathway of common conditions, such as pneumonia and diarrhea.^(1)^ In March 2018, a systematic review was published, assessing the incidence and mortality of sepsis among children. It included 15 scientific articles, mostly from developed countries in the Northern hemisphere. According to the authors, the incidence of sepsis in children was 48 cases/100 thousand people/year, and 22 cases of severe sepsis/100 thousand people/year. The authors also conducted an exploratory estimate according to which 1.2 million cases of sepsis occur every year in children.^(2)^ Tan et al. performed a systematic review including 94 scientific articles, of which half had been produced in developing countries. For them, the odds of a child with severe sepsis dying in a developing country is four times higher than in a developed country.^(3)^

A major issue, particularly in the pediatric population, is that the real epidemiology of sepsis in this population is still unknown.^(4)^ Also, the prevalence is different, when assessing emergency department (ED), inpatient ward, and intensive care unit (ICU) patients.

There are few studies looking into the epidemiology of sepsis in pediatric emergency departments, which serve as the entry door for patients into the hospital.^(5-8)^ There are no Brazilian studies on the epidemiology and treatment of sepsis in pediatric emergency departments.

## OBJECTIVE

Describe the prevalence, clinical characteristics, treatment received (times of onset of antibiotics and fluids) and the outcomes of children with sepsis, severe sepsis, and septic shock, admitted to a pediatric emergency department of a public hospital in Brazil, located in a highly socially vulnerable region.

## METHODS

A retrospective, observational study, conducted between February 2015 and August 2016, at the emergency department of *Hospital Municipal Dr. Moysés Deutsch* (M’Boi Mirim), by the *Centro de Estudos e Pesquisa Dr. João Amorim* (CEJAM) and *Hospital Israelita Albert Einstein* (HIAE). It is a secondary care facility located in the far south of the city of São Paulo (state of SP). At the time of the study, it had 300 beds and served a population of 650 thousand inhabitants, in a highly socially vulnerable area. The pediatric emergency department received, on average, 4,000 low to high-complexity patients per month. At the pediatric emergency department, laboratory and imaging tests were offered, such as X-rays, ultrasonography and computed tomography. It had 20 observation beds, of which two were isolation beds.

The patients’ legal guardians were invited for a meeting, in which the study was explained, and they were asked to sign an Consent Form authorizing access to patient charts. Waiver of Consent Form was requested for patients whose legal guardians did not attend the meeting or were not located, as well as for those who died.

### Criteria and definitions

Sepsis, severe sepsis, and septic shock were defined according to the International Pediatric Sepsis Consensus Conference (IPSCC).^(9)^ In this consensus, systemic inflammatory response syndrome (SIRS) is the presence of at least two of four criteria: fever or hypothermia (temperature >38.5°C or <36°C); tachycardia or bradycardia for the age; tachypnea; leukocytosis, or left-shift. One of the criteria must be abnormal temperature or abnormal leukocyte count. Sepsis is understood as SIRS associated with the presence of suspected or confirmed infection. Severe sepsis means sepsis associated with one of the following factors: cardiovascular dysfunction or respiratory dysfunction, or two other organ dysfunctions. Septic shock is defined as sepsis and cardiovascular dysfunction (non-responsive to fluid therapy and requiring vasoactive drugs).

The treatment criteria used were those of the 2012 Surviving Sepsis Campaign (SSC), which recommends the use of antibiotics and fluid infusion within the first hour after a sepsis diagnosis, as well as the use of vasoactive drugs in case of no improvement after 40 to 60mL/kg of crystalloid fluids.^(10)^

At M`Boi Mirim, there is a Pediatric Sepsis Protocol which provides for collection of a blood culture, arterial gases, and blood lactate levels. The protocol also mandates the use of ceftriaxone for patients from the community; association with clindamycin if toxic shock is suspected; as well as introduction of vancomycin and cefepime for patients with a history of recent hospitalization.

Chronic condition was defined as any medical condition persisting for at least 12 months and requiring specialty care.^(9)^

### Inclusion criteria

This study enrolled all children aged up to 14 years, seen at the emergency department, who met the criteria for sepsis, according to the 2005 IPSCC definitions.^(9)^ Patients were screened through inclusion in the Pediatric Sepsis Protocol used at the hospital and the International Statistical Classification of Diseases and Related Health Problems, 10^th^ edition (ICD-10) for sepsis-related hospital discharge: A41.9 for sepsis, R57 for shock, and A39 for meningococcal meningitis. Patient charts were reviewed by a team of physician and nurses.

The codes adopted were A41.9 and R57, because these were the ICD-10 codes used by the Medical Data Archiving and Statistics Service (SAME) for sepsis and septic shock, respectively.

### Exclusion criteria

Patients who had sepsis, severe sepsis, and septic shock during their stay at the neonatal intensive care unit, the pediatric ICU, and the pediatric inpatient ward were excluded from the study.

### Ethical aspects

The study was approved by the Ethics Committee of the *Secretaria Municipal de Saúde de São Paulo* under CAAE: 53012316.0.0000.0086 and opinion #1.573.174, and by the Research Ethics Committee of *Hospital Israelita Albert Einstein* under CAAE: 53012316.0.3001.0071 and opinion # 1.693.744, respectively.

### Statistical analysis

The variables collected were age, sex, weight, date and time of admission and discharge from the hospital, and date and time of admission and discharge from the pediatric ICU.

Upon admission, the variables collected were presence of cardiorespiratory arrest, heart and respiratory rates, blood pressure, peripheral perfusion data, oxygen saturation, and presence of altered level of consciousness, cyanosis and reduced diuresis.

To assess the treatment, the variables collected were date and time of hospital admission (considered as time point zero) and date and time of onset of crystalloid fluids, antibiotics, and vasoactive drugs. Times were categorized into 1 hour (drugs administered within 1 hour), 2 hours (period from 1 to 2 hours), 3 hours (period from 2 to 3 hours), 4 hours (period from 3 to 4 hours), 5 hours (period from 4 to 5 hours), 6 hours (period from 5 to 6 hours), and more than 6 hours (treatment initiated within more than 6 hours).

The presence of cardiovascular, respiratory, neurological, hematological and hepatic dysfunction was assessed in the form of categorical variables, according to the 2005 IPSCC definitions of dysfunctions.^(9)^

The classifications of sepsis, severe sepsis, and septic shock in patient charts were carried out by one of the authors and by registered nurses from the Quality and Safety Department, previously trained by the author.

The endpoints were discharge and death, number of dysfunctions on evolution, ICU admission, and length of hospital stay.

Categorical variables were reported as absolute and relative frequencies. Numerical variables were described as median and interquartile range, since their distribution was not normal.

The study groups were compared by Fisher’s exact test, for categorical variables, or the Mann-Whitney Test for numerical or ordinal variables.

The analyses were conducted using the R software program (R Core Team, 2019), version 3.1.3.

## RESULTS

**Figure 1:**
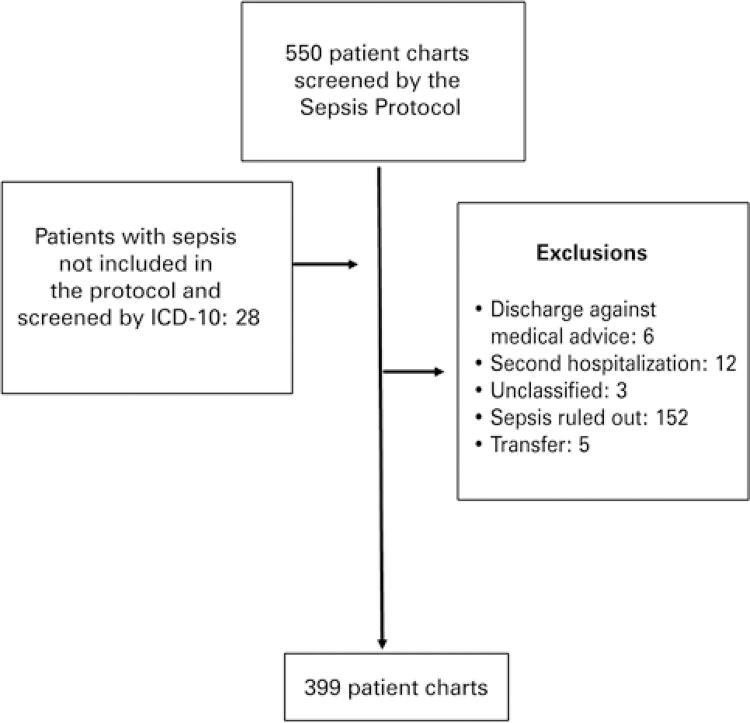
Flow chart of data collection

Between February 2015 and August 2016, a total of 71,174 patients were seen at the emergency department. Of these, 399 patients met the criteria for sepsis, and 371 were screened by the Pediatric Sepsis Protocol and 28 by the ICD-10 (Figure 1).

ICD-10: International Statistical Classification of Diseases and Related Health Problems, 10^th^ edition.

The prevalence of sepsis, severe sepsis, and septic shock at the emergency department was, respectively, 0.41% (n=292), 0.14% (n=97), and 0.014% (n=10).

Table 1 shows the demographics of pediatric patients, the primary conditions associated with sepsis, severe sepsis, and septic shock, and the clinical presentation of each category. The median age was 21.5 months for sepsis, 12 months for severe sepsis, and 20.5 months for septic shock, however with no statistical significance. Males were the most affected sex group. The diagnoses of sepsis, severe sepsis, and septic shock were most frequently associated with respiratory diseases, urinary tract infection, and acute diarrheal disease. An association with chronic conditions was found in 30% (p<0.05) of the septic shock cases.

**Table 1:** Demographics, vital signs upon admission and primary diagnosis

Factors	Sepsis (n=292)	Severe sepsis (n=97)	Septic shock (n=10)	p value
Male	151 (51.9)	50 (51.5)	6 (60)	0.911
Age (months)	21.50 [7.89-48.00]	12.00 [4.00-43.00]	20.5 [2.29-57.02]	0.135
SIRS criteria				
Heart rate				
Bradycardia	0 (0.0)	0 (0.0)	1 (12.5)	0.002*
Normal	84 (28.8)	16 (16.7)	3 (37.5)	
Tachycardia	208 (71.2)	80 (83.3)	4 (50)	
Respiratory rate				
Normal	134 (45.9)	46 (47.9)	3 (33.0)	0.708
Tachypnea	158 (54.1)	50 (52.1)	6 (66.7)	
Clinical criteria for organ failure				
BP measurement on triage	149 (51.0)	59 (60.8)	5 (55.6)	0.233
Skin mottling	15 (5.3)	9 (9.7)	1 (11.1)	0.208
Blood pressure				<0.001*
Hypertension	0 (0.0)	0 (0.0)	1 (20)	
Hypotension	0 (0.0)	17 (29.3)	1 (20)	
Normal	148 (100.0)	41 (70.7)	3 (60)	
Decreased urine output	25 (9.4)	10 (12.2)	1 (12.5)	0.566
Perfusion				0.067
*Flush* (less than 1 second)	16 (5.7)	2 (2.2)	0 (0)	
Slower (more than 3 seconds)	37 (13.2)	12 (13.2)	4 (50)	
Normal	228 (81.1)	77 (84.6)	4 (50)	
Altered level of consciousness	44 (15.5)	25 (26.0)	7 (70)	<0.001*
Decreased oxygen saturation	98 (34.4)	37 (38.5)	4 (44.4)	0.581
Primary conditions				
Pneumonia	89 (30.5)	37 (38.1)	4 (40)	0.307
Bronchiolitis	39 (13.4)	24 (24.7)	0 (0)	0.013*
Meningitis	6 (2.1)	5 (5.2)	1 (10)	0.077
Urinary tract infection	23 (7.9)	11 (11.3)	0 (0)	0.442
Tonsillitis	13 (4.5)	1 (1.2)	0 (0)	0.379
ADD	35 (12)	7 (7.2)	0 (0)	0.300
FWS	27 (9.2)	9 (9.3)	0 (0)	0.876
Asthma	15 (5.1)	4 (4.1)	0 (0)	0.873
Other conditions	52 (17.8)	14 (14.4)	2 (20)	0.655
Chronic disease	19 (6.5)	8 (8.2)	3 (30)	0.043*

* Values with statistical significance.

Results expresses as n (%) or median [1^st^ – 3^rd^ quartiles].

SIRS: systemic inflammatory response syndrome; BP: blood pressure; ADD: acute diarrheal disease; FWS: fever without a source.

There was no combination of common clinical signs in patients with sepsis or severe sepsis.

Blood pressure was measured in barely more than 50% of patients diagnosed with sepsis, severe sepsis, and septic shock. Among patients with severe sepsis, 29.3% had hypotension on admission and, among patients with septic shock, 20% had hypotension on the initial physical examination at the emergency department.

The diagnosis of septic shock, according to the 2005 IPSCC definition, was mostly confirmed by a combination of tachycardia, tachypnea, altered level of consciousness, and abnormal perfusion.

Vital signs measured on admission were missing on some patient charts, which led to differences in the absolute values assessed and the total sum of patients enrolled in the study.

Table 2 shows the agents most frequently isolated. A total of 292 blood cultures were collected. There were 32 positive samples from sepsis, severe sepsis, and septic shock patients, with a 11% positivity rate among samples. A pathogen was isolated in 12% of sepsis cases (95% confidence interval - 95%CI: 9-16.6), 14.4% of severe sepsis cases (95%CI: 8.8-22.8), and 27.3% of septic shock cases (95%CI: 9.7-56.6).

**Table 2:** Pathogens identified

Pathogens	Sepsis (n=292)	Severe sepsis (n=97)	Septic shock (n=10)	p value
Influenza	3 (1.0)	0	0	0.08
Respiratory Syncytial Virus	12 (4.1)	8 (8.2)	1 (10.0)	0.170
Other viruses	0	1 (1.0)	0	0.268
*Streptococcus sp*	3 (1.0)	1 (1.0)	1 (11.0)	0.133
*Staphylococcus sp*	13 (4.5)	1 (1.0)	0	0.379
*Escherichia coli*	5 (1.7)	3 (3.1)	1 (10.0)	0.134
Other bacteria	1 (0.3)	3 (3.1)	0	0.142

Results expressed as n (%).

Among the isolated pathogens, Respiratory Syncytial Virus was the most frequent. The second most frequent was *Staphylococcus sp*. Among the cases in which *Staphylococcus sp* was isolated (14 patients), four blood cultures tested positive for *Staphylococcus epidermidis*; two for *Staphylococcus hominis*, and two for *Staphylococcus aureus*. The other blood cultures had one positive sample for each pathogen: *Staphylococcus vestibularis, Staphylococcus saprophyticus, Staphylococcus arlettae, c*oagulase-negative *Staphylococcus, Staphylococcus capitis*, and *Staphylococcus cohnii*. The pathogen *Streptococcus sp* was isolated in five blood cultures, of which two were positive for *Streptococcus pneumoniae*, one for *Streptococcus pyogenes*, one for *Streptococcus agalactiae*, and one for *Streptococcus salivarius*

Table 3 described the treatment times and outcomes. The median length of hospital stay of patients with sepsis, severe sepsis and septic shock was 3 days, 4 days and 1 day (<0.001), respectively.

**Table 3:** Management and outcomes of sepsis, severe sepsis, and septic shock

Factors	Sepsis (n=292)	Severe sepsis (n=97)	Septic shock (n=10)	p value
Time to fluid administration	3.00 [2.00-5.00]	3.00 [2.00-6.00]	2.00 [1.00-3.00]	0.222
Time to antibiotic administration	3.00 [2.00-6.00]	3.00 [2.00-6.00]	2.50 [1.25-3.75]	0.233
Time to vasoactive drug administration			6.00 [3.00-6.00]	
ICU admission	21 (7.2)	18 (18.8)	7 (70)	<0.001*
Length of ICU stay, days				0.212
Median [1^st^-3^rd^ quartiles]	7 [4-13]	9 [6-13]	1 [1;12,59,75]	0.402
In-hospital death	4 (1.4)	1 (1)	7 (70)	<0.001*

* Statistical significance.

Results expressed as median [1^st^-3^rd^ quartiles] or n (%). p values for Fisher’s exact test and Mann-Whitney test.

ICU: pediatric intensive care unit.

Most patients with sepsis and severe sepsis were discharged from hospital. Most patients with septic shock died. The overall mortality rate was 2%.

## DISCUSSION

To the best of our knowledge, this was the first Brazilian study to assess the prevalence, clinical characteristics, management, and outcome of children with sepsis, severe sepsis, and septic shock in a Brazilian pediatric emergency department. The study also highlights the importance of the emergency department for managing sepsis, since it is the entry door for patients into the hospital, and is the place that most influences patient outcomes, by enabling early treatment and management.

Table 4 shows a summary of epidemiological studies conducted in pediatric emergency departments.

**Table 4:** Summary of epidemiological studies conducted at pediatric emergency departments

Study	Location	Prevalence	Median age	Dysfunctions on admission	Most frequent infection site	Most frequent pathogens	Length of hospital stay (days)	Prevalence of chronic disease (%)	Mortality (%)
Vekaria-Hirani et al.,^(5)^	Tertiary care facility, Nairobi, Kenya	Septic shock (15.4%)	4 months			*Staphylococcus aureus*			9.30
Van de Voorde et al.,^(6)^	Multicenter: 16 emergency departments from 12 countries	Septic shock (0.6%)	2 years	Hypotension (47.7%) and hypoxemia (33.3%)	Pulmonary	*Neisseria meningitidis*	7	35.8	4.50
Kortz et al.,^(7)^	Tertiary care facility, Dar es Salaam, Tanzania	Sepsis (18%)	25 months	Respiratory dysfunction		Malaria (6.9%)	6		14
Ganjoo et al.,^(8)^	Reference hospital, Srinagar, India	Among patients with SIRS: sepsis (14.9%), severe sepsis (3.4%) and septic shock (2.2%)	1 year to 6 years (46% of patients)		Pulmonary	*Staphylococcus aureus*	6.5	4.10	1.90

SIRS: systemic inflammatory response syndrome.

As in other studies, the prevalence of sepsis in the population seen at the emergency department was low (the prevalence of sepsis, severe sepsis, and septic shock was, respectively, 0.41%, 0.14% and 0.014%). However, mortality from septic shock was quite high (70%). There has been an increased incidence of sepsis in recent years, as well as more severe outcomes, in developing countries.^(3,11)^

The prevalence of sepsis can vary based on the different definitions and diagnostic criteria adopted; study design (prospective or retrospective); country or region (level of socioeconomic development, health policies); time of data collection; clinical characteristics and demographics of the study population; data source used; selection bias, and even by hazard. The criteria used for case triage in this study were those of the 2005 Consensus Conference and the ICD-10, enabling a comprehensive search for patients with these conditions.^(12)^

The prevalence in this study was similar to those observed in the Research in European Pediatric Emergency Medicine (REPEM) study, in which the prevalence of sepsis and severe sepsis was 0.6%.^(6)^In a study by Singhal et al., the prevalence of severe sepsis in emergency departments in the U.S. was 0.35%.^(13)^ Kortz et al. found a 2% prevalence of sepsis.^(7)^

These findings are different from those of Ganjoo et al.^(8)^ and Vekaria-Hirani et al.^(5)^ The first investigated only patients meeting the SIRS criteria, and the prevalence of sepsis, severe sepsis and septic shock was 14.9%, 3.5% and 2.1%, respectively.^(8)^ For Vekaria-Hirani et al. the prevalence of septic shock was 15.4%. This higher prevalence can result from the management during transportation of patients from other services to the hospital, and the long trip to the reference hospital.^(5)^

Data collection in this study took place during the process of implementing the sepsis protocol at the hospital. In this study, most cases were included in said protocol. Training for protocol implementation and the protocol itself may have helped in applying the IPSCC criteria in daily practice, and may be a strategy for bridging the gap between definitions and clinical practice.

The findings of this study point to predominance of male sex, and median age of 22.5 months for sepsis, 13 months for severe sepsis, and 13.10 months for septic shock. These data are similar to those of Kortz et al.^(7)^ and Vekaria-Hirani et al.^(5)^ For Kortz et al. the median age of sepsis patients was 25 months, and 73% of patients were under 5 years old.^(7)^ In Vekaria-Hirani et al., the median age of septic shock patients was 4 months.^(5)^ In the REPEM study, the median age was 4 years.^(6)^

Blood pressure was measured in marginally more than 50% of patients. In these cases, hypotension was present in 29.3% of patients with severe sepsis and 30% of patients with septic shock. This difficulty was also found by Vekaria-Hirani et al., who verified that only 5% of patients had their blood pressure measured.^(5)^ Despite the small numbers, these data corroborate that hypotension can be a late sign of heart dysfunction in pediatric patients, and it must be diagnosed based on other signs from the physical examination, such as altered level of consciousness and abnormal perfusion.^(14)^ Measuring the blood pressure in children is more difficult, because it requires appropriate size cuff and patients’ collaboration.^(15)^

The most common dysfunctions at triage were respiratory and neurological dysfunctions. This was also found in the REPEM study.^(6)^ In Kortz et al., respiratory failure was the most common dysfunction.^(7)^ It is possible that the higher rate of respiratory failure is reflecting the reality of an emergency department at a secondary care facility, which sees a large number of patients with respiratory diseases. Also, in many cases, it is difficult to differentiate sepsis and respiratory failure, because they share clinical signs, such as tachypnea, hypoxemia and abnormal tissue perfusion. This issue was also described by Weiss et al., in a study that found a low level of agreement between clinical definitions, research definitions, and the International Classification of Diseases, Clinical Modifications (ICD-9-CM).^(16)^ Therefore, it is possible that, at early implementing stages of the sepsis protocol, patients with respiratory failure due to bronchiolitis and asthma may have been diagnosed with sepsis and managed accordingly after an initial evaluation.

The pathogen most frequently isolated in this study was the Respiratory Syncytial Virus, differently from other studies, in which bacteria were most common. In the studies by Ganjoo et al. and Vekaria-Hirani et al., *Staphylococcus sp* was the most frequent, and in Kortz et al., a large part of patients had malaria.^(5,7,8)^The low number of patients with positive blood cultures raises the hypothesis that their sepsis may have been of viral origin. Lin et al. argued the epidemiology of viral sepsis is unknown, because many epidemiological studies did not look into data on viral infections.^(17)^ In an epidemiological study in Southeast Asia, viral agents were isolated in 76% of children with sepsis.^(18)^

The most common diagnoses were pneumonia and bronchiolitis. This data help understand the predominance of respiratory dysfunctions at triage and identification of the Respiratory Syncytial Virus as the most frequent pathogen.

In this study, there was association with chronic diseases in 33% of septic shock cases, very similar to the finding of the REPEM study, 35.8%.^(6)^

As for the management, it was difficult to compare the findings of this study with others, because there is no consensus in the literature for definition of time point zero.^(19)^ In this study, we adopted as time point zero the time of admission of the patient – and not the time of diagnosis, as seen in Paul et al.^(20)^ and Fernández-Sarmiento et al.^(21)^ For Paul et al., the median time to fluid administration was 83 minutes, and 88 minutes for antibiotics.^(19)^ In Fernández-Sarmiento et al., the time to fluid administration was 5 minutes, and the time to antibiotics was 271 minutes.^(21)^

The median length of stay in this study was 3, 4 and 1 days for sepsis, severe sepsis, and septic shock, respectively. These values are lower than those found by Kortz et al., Ganjoo et al. and the REPEM study.^(6-8)^ In patients with septic shock, the short length of stay may be due to late arrival at the health facility. In patients with sepsis and severe sepsis, there are two possible explanations: the length of stay may be due to good response to early treatment, resulting in prevention of dysfunction and hospital discharge after stabilization and clinical improvement, switching to oral antibiotics; or patients with sepsis-like clinical conditions (such as respiratory failure and febrile illness) who may have been diagnosed and treated for sepsis.

The mortality in this study was 3%, similar to the findings of Ganjoo et al. and the REPEM study.^(6,8)^ The mortality in this study is also lower than those of other studies in pediatric ICUs.^(22-24)^ This mortality rate is believed to reflect the reality of an emergency department at a secondary care facility, which receives a high number of low-complexity patients. Also, ICU patients are more severe and more likely to die.

Patients who died had a shorter length of stay and already presented with different organ dysfunctions on admission. This was also seen in the publications by Kortz et al. and Vekaria-Hirani et al.^(5,7)^ In Vekaria-Hirani et al., most patients died within the first five hours.^(5)^ In Kortz et al., 10.5% of patients died before admission to the ICU, and the median time between admission and death was 3 days.^(7)^ These data support late arrival to the hospital. Launay et al. looked into the causes of death in children with severe bacterial infections. The delay in health-care seeking by parents was one of the barriers for proper management.^(25)^ It is believed that educating parents on what sepsis is and how to recognize it is a major step towards early sepsis treatment. The first hour of sepsis very often takes place at home, and not at a hospital.

This study has some limitations. This is a retrospective study, and some data are missing due to missing information in patient records. It is difficult to diagnose sepsis in the pediatric setting, and the prevalence may be underestimated due to encouragement of protocol use. The study depicts the reality of one site and may not reflect the reality of other regions in Brazil. ICD codes for infrequent conditions were not used for sepsis, severe sepsis, and septic shock triage, such as actinomycotic sepsis (A42.7), anthrax sepsis (A22.7), toxic shock syndrome (A48.3), listerial sepsis (A.32.7), disseminated herpes virus disease (B00.7) and candidal sepsis (B37.7). Discharge and death locations (emergency department or inpatient ward) of patients were not assessed. Nutritional status and vaccination status of patients were not assessed, neither was the socioeconomic status of parents. The severity score was not used for patients on admission to the emergency department. This was one difficulty found: being able to apply the mortality score to all patients, not just those admitted to the ICU. The Pediatric Risk of Mortality (PRISM) and Pediatric Index of Mortality (PIM) scores and versions have been validated to be used in pediatric ICUs.^(26-29)^ In emergency departments, triage scores, such as the Manchester, the Canadian Triage and Acuity Scale (CTAS), the Australian Triage Scale (ATS) and the South African Triage Scale (SATS) were predictive of mortality in children.^(30)^ In the study hospital, the triage score was not validated for mortality.

Brazil is the fifth most populous country, with major economic and social differences between its regions. More epidemiological and multicenter studies are needed to provide appropriate data for guiding effective policies.

## CONCLUSION

Sepsis was not of the most prevalent conditions in this pediatric emergency department. However, mortality from septic shock was high. The most common dysfunctions at triage were respiratory and neurological dysfunctions. The median time to treatment onset was 3 hours. The emergency department is the entry door to the hospital, and a diagnosis of sepsis poses a challenge to the emergency department staff: identifying signs of severe disease among several children with fever, frequent diseases with sepsis-like presentations (asthma and bronchiolitis), and different complaints that do not require urgent management.
